# Characterization of erythroferrone oligomerization and its impact on BMP antagonism

**DOI:** 10.1016/j.jbc.2023.105452

**Published:** 2023-11-09

**Authors:** Eleanor M. Mast, Edmund A.E. Leach, Thomas B. Thompson

**Affiliations:** Department of Molecular and Cellular Biosciences, University of Cincinnati, Cincinnati, Ohio, USA

**Keywords:** iron, bone morphogenetic protein (BMP), analytical ultracentrifugation, molecular modeling, mutagenesis, protein purification, surface plasmon resonance (SPR), erythroferrone, transforming growth factor beta family (TGF-β), C1Q/TNF-related protein family (CTRP)

## Abstract

Hepcidin, a peptide hormone that negatively regulates iron metabolism, is expressed by bone morphogenetic protein (BMP) signaling. Erythroferrone (ERFE) is an extracellular protein that binds and inhibits BMP ligands, thus positively regulating iron import by indirectly suppressing hepcidin. This allows for rapid erythrocyte regeneration after blood loss. ERFE belongs to the C1Q/TNF-related protein family and is suggested to adopt multiple oligomeric forms: a trimer, a hexamer, and a high molecular weight species. The molecular basis for how ERFE binds BMP ligands and how the different oligomeric states impact BMP inhibition are poorly understood. In this study, we demonstrated that ERFE activity is dependent on the presence of stable dimeric or trimeric ERFE and that larger species are dispensable for BMP inhibition. Additionally, we used an *in silico* approach to identify a helix, termed the ligand-binding domain, that was predicted to bind BMPs and occlude the type I receptor pocket. We provide evidence that the ligand-binding domain is crucial for activity through luciferase assays and surface plasmon resonance analysis. Our findings provide new insight into how ERFE oligomerization impacts BMP inhibition, while identifying critical molecular features of ERFE essential for binding BMP ligands.

Erythroferrone (ERFE) is the primary erythroid regulator of iron metabolism (1). When iron overload is sensed, bone morphogenetic protein (BMP)-induced hepcidin transcription occurs in hepatocytes, which negatively regulates gut iron import (2, 3, 4). After a blood loss event, erythroid precursor-produced ERFE directly antagonizes BMP ligands (1, 5, 6). By turning off this negative regulatory mechanism, ERFE positively regulates iron import, allowing for rapid regeneration of erythrocytes. ERFE contributes to iron overload in a mouse model of β-thalassemia which, like human patients, experience pathogenic erythropoiesis and iron overload (7, 8, 9). Both liver and spleen iron overload in these mice was partially alleviated after treatment with an ERFE neutralizing antibody or genetically by crossing β-thalassemic mice with ERFE^−/−^ mice (7, 8). Thus, a better understanding of ERFE and its mechanism of action may lead to a better comprehension of the role of ERFE in pathophysiological states of iron regulation.

ERFE is part of the 16-member C1Q/TNF-related protein (CTRP) family. While ERFE is the only member known to antagonize BMP ligands, it shares the family's characteristic form (Fig. 1*A*) (10). CTRPs are named for their C-terminal globular C1Q domain (10); numerous crystal structures of C1Q domains depict a TNF-α-like trimer held together by noncovalent interactions (11, 12, 13). CTRPs also possess an N-terminal unstructured domain (USD) containing unpaired cysteines and a proline/glycine-rich collagen-like repeat (CLR) which, like collagen, is predicted to form a triple helix (10, 13, 14, 15). Well-characterized family members are known to trimerize using their C1Q and CLR and form higher order multimers by linking trimeric units together with intermolecular disulfide bonds (16, 17, 18). While published data suggest ERFE may oligomerize similarly (19, 20), the link between ERFE oligomerization and activity remains unexplored.

**Figure 1:**
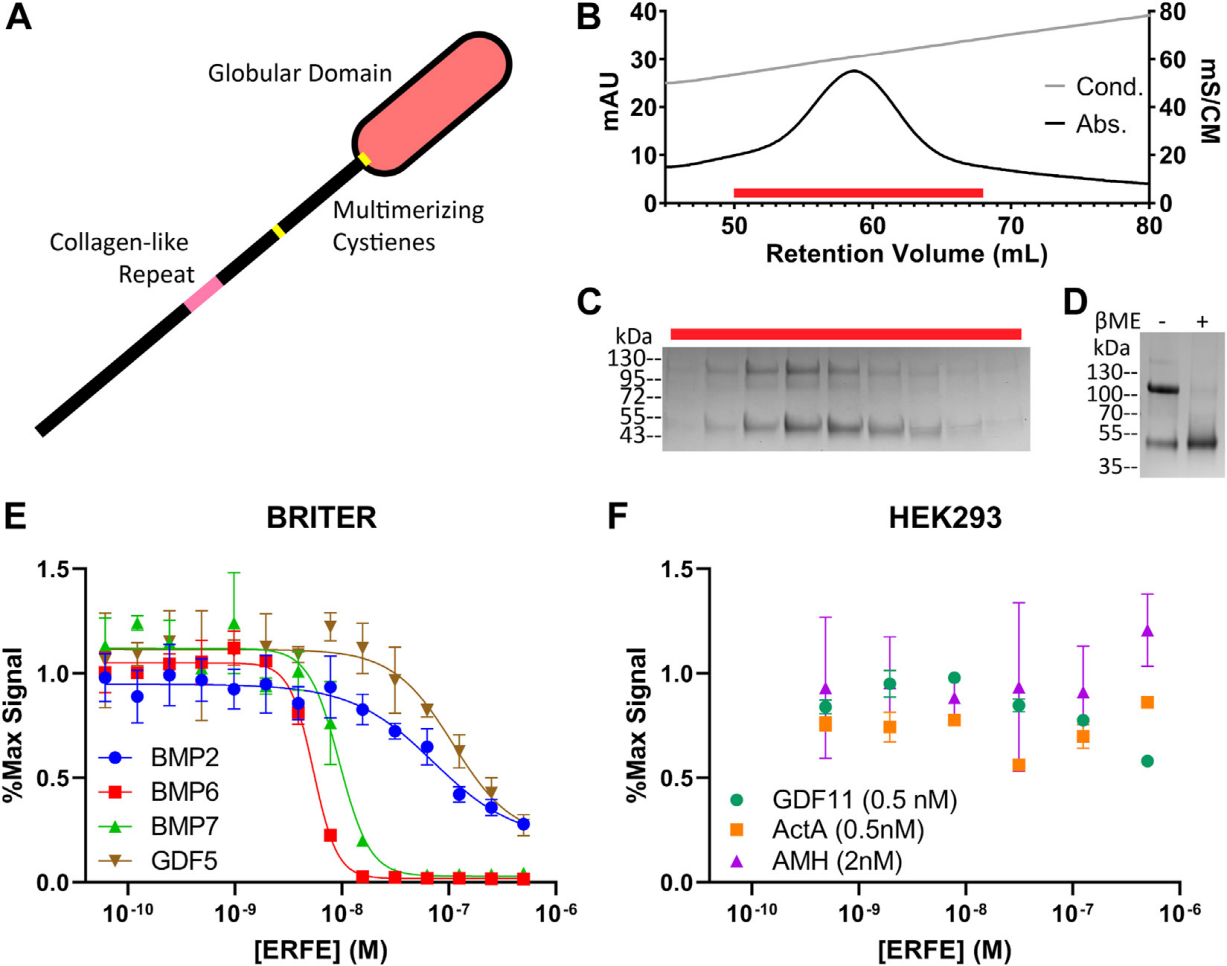
**Purified ERFE is a BMP-specific antagonist.***A*, schematic of with the C1q domain shown as an *oval*. The USD contains the collagen-like repeat and cysteine predicted to aid in oligomerization. *B*, WT ERFE purified using heparin affinity chromatography and eluted with a salt gradient. *C*, peak fractions corresponding to the *red bar* were analyzed *via* SDS-PAGE. *D*, purified ERFE analyzed under reducing and nonreducing conditions. ERFE contains a disulfide-linked dimer and monomer component. *E* and *F*, ERFE inhibition was measured *via* BMP responsive luciferase assay using a BRE promoter. ERFE was titrated against 2.5 nM BMPs in BRITER cells (n = 3 biological replicates, representative curve shown) and different concentrations of other ligands (n = 2 biological replicates, representative curve shown) in HEK293 cells, all starting at 500 nM ERFE. Statistical analysis is detailed in Experimental procedures and results are in Table 1. SD of three points within a single technical replicate shown with error bars. BMP, bone morphogenetic protein; ERFE, erythroferrone.

ERFE is known to bind and inhibit BMPs, which are the largest subfamily of the transforming growth factor-β (TGF-β) family of signaling ligands (6). Like all TGF-β ligands, BMPs signal by binding to a pair of type I receptors and a pair of type II receptors, leading to receptor activation and SMAD 1/5/9-dependent changes in DNA transcription (21, 22). BMPs are heavily regulated by a number of structurally diverse extracellular antagonists which tightly bind to BMPs, occluding their receptor binding sites (21, 23, 24). These antagonists bind with varying specificity across the subfamily of BMP ligands and are in large part driven by distinct binding mechanisms. These mechanisms include differences in binding stoichiometry—chordin (monomer) and noggin (dimer) both bind BMP ligands in a 1:1 ratio, whereas gremlin-2 (dimer) binds in a 2:1 ratio (24, 25, 26). ERFE, a trimeric inhibitor, represents a potentially unique and uncharacterized binding modality found within BMP antagonists.

In this study, we demonstrated that ERFE potently inhibited BMP subfamily members BMP6 and BMP7 and inhibited BMP2 and GDF5 to a lesser degree. We determined the functional inhibitory unit of ERFE is a trimer, as oligomeric states above a trimer did not differ in BMP6 inhibition and states below trimer were less efficacious. Finally, we used AlphaFold modeling and cross-species conservation analysis to identify a highly conserved helix in ERFE’s USD that was predicted to bind in the same location as type I receptors. By utilizing site-directed mutagenesis and surface plasmon resonance (SPR), we provide evidence that a conserved helix within the USD, which we termed the ligand-binding domain (LBD), is critical for BMP antagonism by inhibiting BMP:type I receptor interaction.

## Results

### Isolation of ERFE—A BMP 5/6/7-specific antagonist

**Supporting Figure S1:**
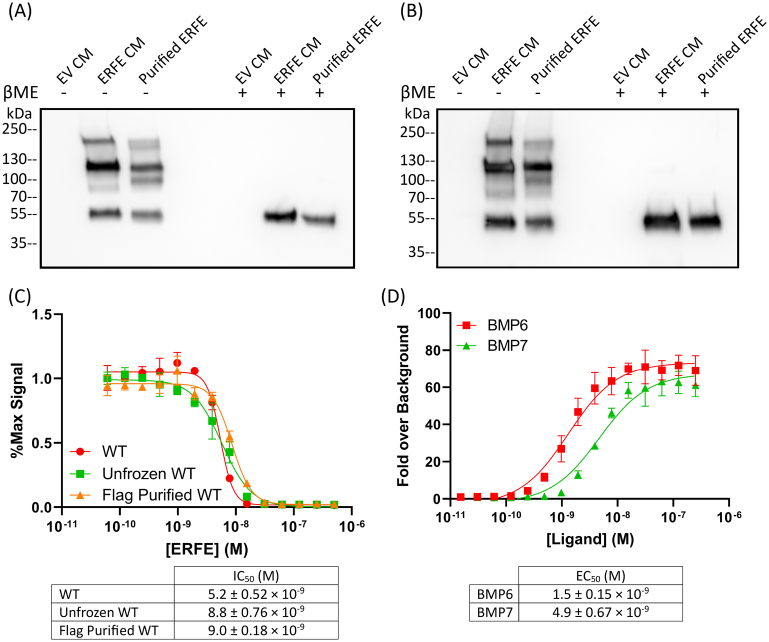
**Verification of reagents and purification methods.***A* and *B*, purified ERFE was probed in western blot with (*A*) αERFE or (*B*) αFLAG-tag antibodies. *C*, WT ERFE did not change its potency regardless of freezing or purifying *via* flag affinity followed by heparin affinity chromatography. *D*, BMP6 and BMP7 used in this paper are potent activators of BMP signaling in BRITER cells (n = 3 biological replicates, representative curve shown). Statistical analysis is detailed in Experimental procedures; data in the table represent the mean IC_50_ of three independent experiments ± SEM. BMP, bone morphogenetic protein; ERFE, erythroferrone.

Previous studies reported that ERFE potently inhibits BMP5, BMP6, and BMP7, but does not strongly inhibit BMP2, BMP4, BMP9, or Activin B (6, 8, 27, 28). To investigate the molecular basis for this inhibition, we isolated N-Flag ERFE *via* heparin affinity chromatography (Fig. 1*B*). The resulting protein was >95% pure when analyzed *via* SDS-PAGE, and all bands were confirmed to include ERFE as shown by both anti-flag and anti-ERFE western blots (Figs. 1*C* and S1, *A* and *B*). The majority of ERFE appeared primarily in two distinct bands with molecular weight corresponding to a monomer and a disulfide-linked dimer, as was previously reported (19) (Fig. 1*D*). Consistent with previous results, ERFE strongly inhibited BMP6 and BMP7, while less potently inhibiting BMP2 signaling in a cell-based luciferase reporter assay. We also tested other ligands, including GDF5, Activin A, GDF8, and anti-Müllerian hormone (Fig. 1, *E* and *F* and Table 1). Only GDF5 showed sensitivity to ERFE, albeit at a reduced level comparable to BMP2. This agrees with previous reports that ERFE is a BMP-specific inhibitor and provides additional evidence of interactions with the GDF5/6/7 subfamily of BMP ligands (6, 8, 28).

**Table 1:** ERFE IC_50_ values when tested against an array of TGF-β ligands

Ligand	IC_50_ (M)
BMP2 (2.5 nM)	1.7 ± 0.57 × 10^−7^
BMP6 (2.5 nM)	5.2 ± 0.52 × 10^−9^
BMP7 (2.5 nM)	8.9 ± 0.75 × 10^−9^
GDF5 (2.5 nM)	1.3 ± 0.25 × 10^−7^
Activin A (0.5 nM)	n/a
GDF11 (0.5 nM)	n/a
AMH (2 nM)	n/a

IC_50_ is the average of three biological replicates, ±SEM.

### Collagen-like repeat- and cysteine-mediated oligomerization is dispensable for ERFE inhibition of BMP signaling

**Figure 2:**
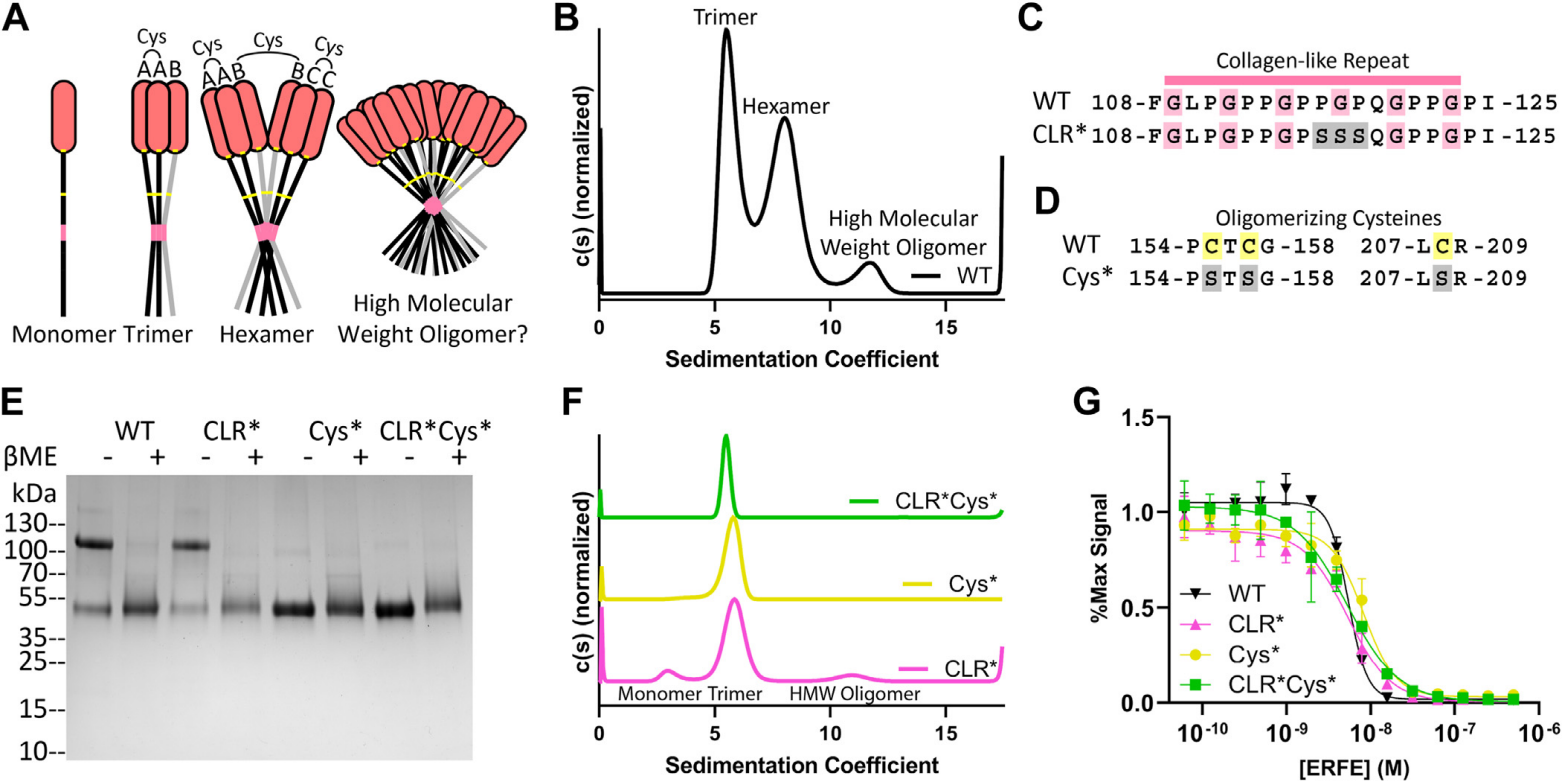
**Collagen-like repeat- and cysteine-mediated oligomerization is dispensable for ERFE inhibition of BMP6.***A*, ERFE is predicted to form higher order oligomers with a trimer as the basic unit, held together by intermolecular disulfide bonds as indicated. *B*, sedimentation velocity reveals that WT ERFE is predominantly trimer and hexamer, with a small fraction of high molecular weight oligomer. *C* and *D*, schematic of two mutants generated to disrupt ERFE oligomerization—CLR∗ disrupts the collagen-like repeat and Cys∗ mutates oligomerizing cysteines into serines. *E*, 5ug purified ERFE mutants were analyzed *via* SDS page under nonreducing and reducing conditions. WT ERFE from Figure 1*D* provided for reference. *F*, sedimentation velocity of analysis of the Cys∗ and CLR ERFE mutants. *G*, analysis of BMP inhibition of the mutants as compared to WT ERFE. BRITER cells were stimulated with 2.5 nM BMP6 and inhibited with increasing concentrations of ERFE and mutations. Statistical analysis is detailed in Experimental procedures and results are in Table 2. SD of three points within a single technical replicate shown with error bars (n = 3 biological replicates, representative curve shown). BMP, bone morphogenetic protein; CLR, collagen-like repeat; ERFE, erythroferrone.

Present studies suggest that ERFE trimerizes and, through intertrimer linkages, forms a hexamer and a high molecular weight oligomer of an unspecified size (Fig. 2*A*) (19, 20). Previous studies relied on analyzing unpurified ERFE *via* blue native-PAGE and detection by Western blot, which yielded indistinct bands that varied between studies (19, 20). To determine the oligomeric forms of ERFE in a more native state, we used sedimentation velocity analytical ultracentrifugation (AUC), a robust biophysical method for protein oligomeric analysis. Inconsistent results were seen when using frozen ERFE, potentially due to variations in freezing and thawing. Thus, all AUC experiments were completed using unfrozen, freshly purified protein. As the predicted frictional ratio of each c(s) peak diverged, c(s) curve fitting was performed starting at the most frequently occurring frictional ratio. Different protein populations had different frictional ratios; therefore, it was impossible to simultaneously solve for their precise molecular weights. This led to discrepancies between expected and observed weights. Consequently, approximate molecular masses were used to assign oligomeric states in a qualitative manner. Native ERFE was revealed to be primarily trimer (∼44%, ∼106 kDa) and hexamer (∼48%, ∼181 kDa) with a small amount of a high molecular weight species (∼7%, ∼319 kDa) (Fig. 2*B*). No other higher-order oligomer was observed in substantial quantities.

Based on the literature surrounding ERFE and other CTRPs (13, 18, 19, 20), we hypothesized that ERFE's CLR stabilized trimeric ERFE and cysteines enabled intertrimer disulfide bridges, allowing for higher order covalent oligomerization (Fig. 2*A*). To interrogate the contributions of each component to oligomerization and BMP inhibition, we created two mutant forms of ERFE. First, we mutated the center PGP in the CLR to SSS (termed CLR∗) (Fig. 2*C*). We predicted this would disrupt formation of a collagen triple helix without causing the decreased production of ΔCLR ERFE as reported by Stewart *et al.* (20) To disrupt intermolecular disulfide bonds, Cys155, 157, and 208—which were previously reported oligomerizing cysteines (20)—were mutated to serines (termed Cys∗) (Fig. 2*D*). Both variations were combined to create a third mutant (CLR∗Cys∗). After production and purification, we analyzed the ERFE mutant *via* SDS-PAGE. We observed that CLR∗ had a similar ratio of disulfide linked dimer to WT ERFE, suggesting this mutation did not affect intermolecular disulfide bonds. As expected, Cys∗ and CLR∗Cys∗ appeared as a single species in nonreducing conditions (Fig. 2*E*).

Intriguingly, as determined by sedimentation velocity, all ERFE mutants were deficient in oligomerization above a trimer (Fig. 2*F*). However, all mutants formed trimers, suggesting the C1Q domain is the main driver in stabilizing trimeric ERFE. As expected, Cys∗ and CLR∗Cys∗ do not form higher order oligomers, as they lack the requisite cysteines. Interestingly, analysis of CLR∗ ERFE by SDS-PAGE reveals a mixture of disulfide-linked dimer and monomer, yet sedimentation velocity analysis reveals minimal oligomerization above a trimer. This suggests that both the CLR and intermolecular disulfide bridges play a role in coordinating ERFE oligomerization.

Previous studies have shown that adiponectin, a well-studied CTRP family member, exhibits different activity and functions based on differences in its oligomeric states (13, 18). Thus, we wanted to determine if reducing the higher-order oligomers of ERFE had an impact on BMP inhibition. As such, we tested the activity of CLR∗, Cys∗, and CLR∗Cys∗ ERFE using a BMP inhibition luciferase assay (Fig. 2*G* and Table 2). The results show that all three mutants had similar IC_50_ values toward BMP6 inhibition when compared to WT ERFE. This suggests that ERFE oligomerization above a trimer is dispensable for BMP inhibition.

**Table 2:** ERFE oligomer-deficient mutant IC_50_ values when tested against 2.5 nM BMP6

ERFE Mutant	IC_50_ (M)	Fold decrease from WT
CLR∗	6.1 ± 0.12 × 10^−9^	1.2
Cys∗	8.7 ± 0.85 × 10^−9^	1.7
CLR∗Cys∗	4.4 ± 0.75 × 10^−9^	0.9
ΔC1Q	7.8 ± 0.53 × 10^−9^	1.5
CLR∗ΔC1Q	2.7 ± 0.17 × 10^−8^	5.2
Cys∗ΔC1Q	9.7 ± 0.67 × 10^−8^	19
CLR∗Cys∗ΔC1Q	2.9 ± 0.11 × 10^−7^	56

IC_50_ is the average of three biological replicates, ±SEM.

**Table 3:** ERFE Δhelix and LBD mutant IC_50_ values when tested against 2.5 nM BMP6

ERFE Mutant	IC_50_ (M)	Fold decrease from WT
ΔH1 (ΔLBD)	n/a	n/a
ΔH2	9.1 ± 2.6 × 10^−9^	1.8
W82A	n/a	n/a
F85A	n/a	n/a
D90A	1.3 ± 0.15 × 10^−8^	2.5
N94A	2.1 ± 0.26 × 10^−8^	4.0

IC_50_ is the average of three biological replicates, ±SEM.

### Ablation of ERFE trimerization decreases inhibition of BMP6

**Figure 3:**
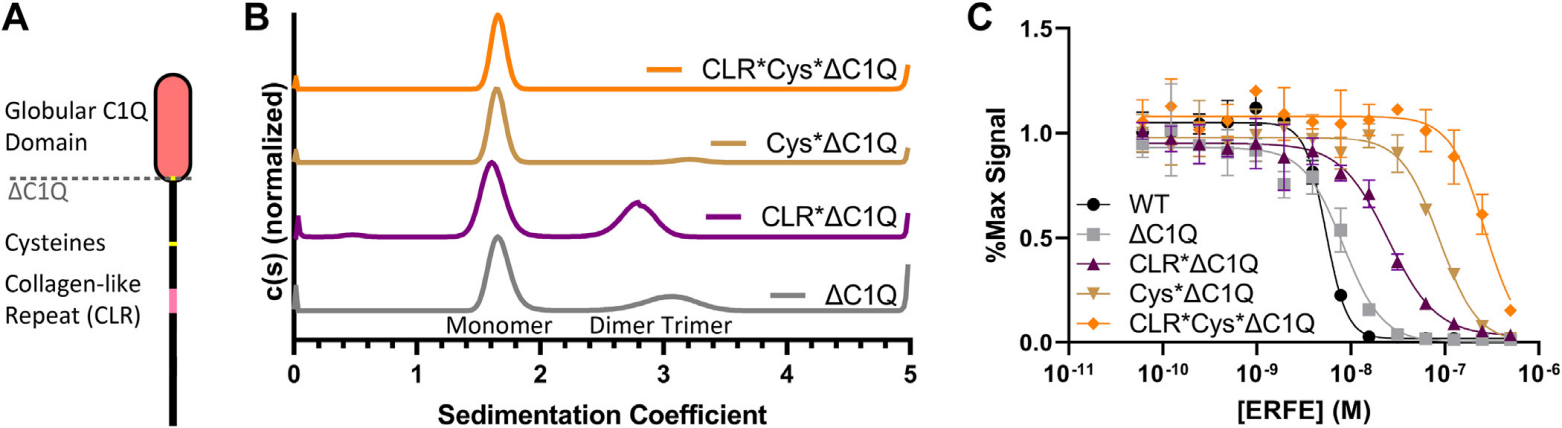
**Ablation of ERFE trimerization decreases inhibition of BMP6.***A*, removal of ERFE's C1Q domain to generate the ΔC1Q mutant. *B*, sedimentation velocity shows that ΔC1Q ERFE had reduced levels of trimer, CLR∗ΔC1Q formed only monomer and dimer, dimer-deficient Cys∗ΔC1Q exhibited minimal trimerization, and CLR∗Cys∗ΔC1Q was entirely monomeric. *C*, BMP reporter assay to measure BMP6 (2.5 nM) inhibition in BRITTER cells of the ERFE mutations. Statistical analysis is detailed in Experimental procedures, and results are in Table 2. SD of three points within a single technical replicate shown with error bars. (n = 3 biological replicates, representative curve shown). BMP, bone morphogenetic protein; CLR, collagen-like repeat; ERFE, erythroferrone.

**Supporting Figure S2:**
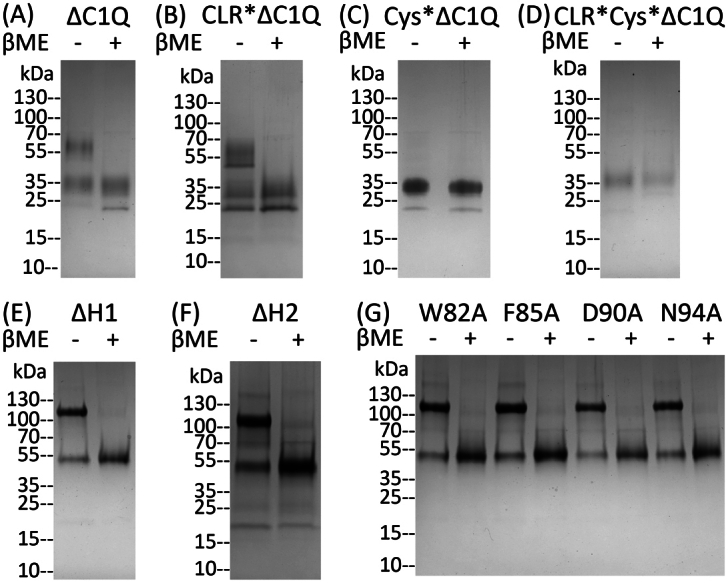
**Verification of produced mutant purity.***A*–*D*, SDS-PAGE was used to analyze protein purity by loading 5ug WT ERFE or the specified ΔC1Q mutant and staining with Coomassie blue. *E*–*G*, the same was done for removal of H1, H2, and ERFE LBD mutants. ERFE, erythroferrone; LBD, ligand-binding domain.

Given its similarity to other CTRPs, ERFE trimerization has been proposed to be driven in part by its C1Q domain. Thus, in order to test if ERFE trimerization is important for BMP inhibition, we hypothesized that truncating the C1Q domain of ERFE would allow for the isolation and characterization of a monomeric form of ERFE. Here, we introduced a stop codon at C208, removing the entire C1Q domain (ΔC1Q) (Fig. 3*A*). The ΔC1Q mutant was combined with the previous mutant schemata (CLR∗ and Cys∗) to generate CLR∗ΔC1Q, Cys∗ΔC1Q, and CLR∗Cys∗ΔC1Q ERFE. Each of these four mutants was expressed and purified in Expi293 cells. Purity was assessed *via* SDS-PAGE, with bands migrating at the expected mass for all constructs (Fig. S2, *A*–*D*). Both ΔC1Q and CLR∗ΔC1Q were composed of disulfide-linked dimer and monomer, while Cys∗ΔC1Q and CLR∗Cys∗ΔC1Q were monomeric.

The four mutants with the C1Q domain removed were analyzed *via* sedimentation velocity (Fig. 3*B*), and molecular mass determination was performed as previously mentioned above. Interestingly, ΔC1Q did not completely disrupt trimer formation and presented as ∼67% monomer (∼23 kDa) and ∼32% trimer (∼57 kDa). The residual trimer was removed by also disrupting the CLR, as CLR∗ΔC1Q was composed of ∼61% monomer (∼19.2 kDa) and ∼38% dimer (∼43.3 kDa) with no trimer present. Surprisingly, Cys∗ΔC1Q was predominantly monomer (∼20.7 kDa) with an exceedingly low amount of trimer (<8%, ∼61.6 kDa). No dimer was present due to the lack of intermolecular disulfide bonds. Finally, CLR∗Cys∗ΔC1Q was entirely monomeric. These data suggest that the C1Q, CLR, and cysteines all contribute to stability of the trimeric form of ERFE.

Next, we wanted to examine whether complete ablation of noncovalently associated trimer (CLR∗ΔC1Q), disulfide-linked dimer (Cys∗ΔC1Q), or reduction to monomer (CLR∗Cys∗ΔC1Q) would change ERFE inhibition of BMP signaling activity (Fig. 3*C* and Table 2). Intriguingly, despite a reduction in the proportion of trimer, ΔC1Q had similar activity to WT ERFE. Ablation of trimeric ERFE, leaving monomer and dimer alone (CLR∗ΔC1Q), decreased activity by 5-fold. Cys∗ΔC1Q, which was predominantly monomer with a small quantity of trimer, was 10-fold less active than WT. Entirely monomeric CLR∗Cys∗ΔC1Q was the least active and had >50-fold less activity than WT ERFE. Taken together, these data suggest ERFE inhibition is driven by stable trimeric or dimeric ERFE.

### Identification and mutagenesis of a LBD in ERFE

**Figure 4:**
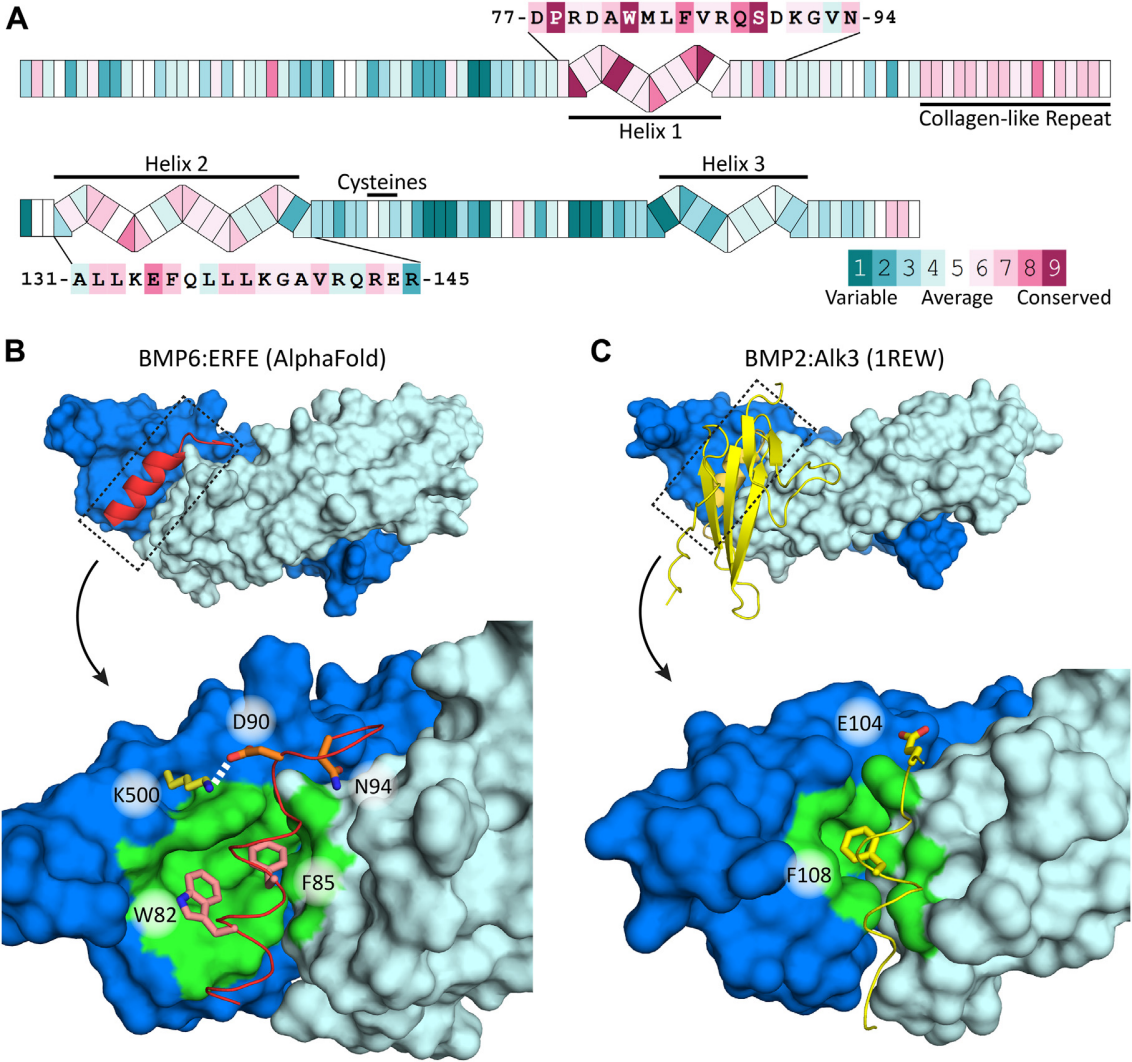
**Cross-species conservation and AlphaFold molecular modeling predicted a highly conserved helix interacts with BMP ligands.***A*, ConSurf analysis of ERFE USD. Two conserved helices (H1, H2) are labeled and sequences shown. Secondary structure predictions were derived from the publicly available AlphaFold structure database (55). *B*, AlphaFold multimer model depicting only the H1 of ERFE bound to BMP6 (chain *A* and *B* are *colored dark* and *light blue*, respectively), (lower) close-up view of H1 interactions with BMP6 showing W82, F85, D90, and N94 of ERFE interacting with the type I pocket of BMP6. *C*, type I receptor:BMP interactions. For both (*B* and *C*), bulky, hydrophobic residues are placed into the hydrophobic pocket to form a “knob-in-hole” motif and residues within 5 Å of W82/F85 (ERFE) and F108 (Alk3) are *colored green*. BMP, bone morphogenetic protein; ERFE, erythroferrone; USD, unstructured domain.

**Supporting Figure S3:**
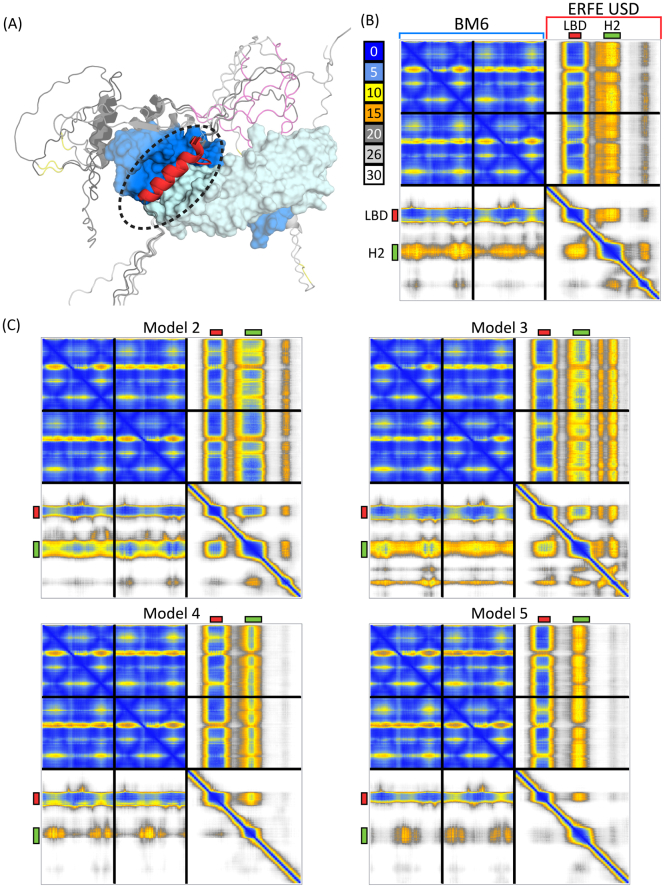
**Evaluation of the five AlphaFold-generated models.***A*, AlphaFold was used to generate five amber relaxed models, all of which were overlayed. H1 (*red*) was the only consistently placed element. The cysteines (*yellow*), CLR (*pink*), and H2 (*black*/*gray*) were never consistently placed. *B*, predicted aligned error (PAE) plot of the highest ranked AlphaFold model generated, which is used in Figure 4*B*. *C*, AlphaFold-generated PAE plots for models ranked 2 to 5. CLR, collagen-like repeat.

Removal of the C1Q domain had little impact on BMP inhibition, supporting previously published data implicating ERFE’s USD as the main inhibitory component (8, 27). In order to gain insight into potential binding mechanisms that enable the USD to bind and inhibit BMP ligands, we used a combination of evolutionary analysis coupled with molecular modeling. First, we used ConSurf to identify which segments in ERFE’s USD are evolutionarily conserved (29, 30). We identified two conserved helices (H1 and H2) on either side of the CLR; the sequence for the more highly conserved N-terminal helix (H1) and less conserved C-terminal helix, (H2) are shown (Fig. 4*A*). Next, we used AlphaFold Multimer (31, 32) to model ERFE's USD with a BMP6 dimer. Strikingly, in all five relaxed models generated, H1 was consistently placed in the BMP6 type I receptor site with high confidence (Fig. S3, *A*–*C*). H2 varied greatly between models and was scored with higher predicted aligned error.

Upon closer examination, the H1:BMP6 interface buries approximately 750 Å^2^ on each component, highlighted by two conserved hydrophobic residues, W82 and F85. The predicted H1 interactions with BMP6 resembled that of other ligand-binding partners (such as receptors, coreceptors, and antagonists), which increased our confidence in the model. For example, the hydrophobic interactions mirror that of type I receptor binding to BMP ligands (BMP2:ALK3 complex, PDB:1REW) (Fig. 4, *B*–*C*) (33). Here, both utilize the common “knob-in-hole” binding motif where a bulky hydrophobic residue on either the H1 (F85) or type I receptors is inserted into a hydrophobic pocket on the ligand surface (22, 34). Intriguingly, AlphaFold also predicted ERFE W82 interacts with two conserved tryptophans located in the concave curvature of the ligand, along with other hydrophobic residues in the ligand pocket. This interaction extends the hydrophobic interface beyond what is seen in receptor knob-in-hole interactions, broadening it toward the tip of the ligand and burying a total of 270 Å^2^ for just W82 and F85. In addition to the central hydrophobic interactions, ERFE was predicted to form ancillary interactions, such as a salt bridge from D90 of ERFE to K500 located in finger 4 of BMP6 (Fig. 4*B*). This interaction resembles the capping interaction seen in other BMP:Noggin interactions (26). Further, N94 of ERFE is predicted to interact with the main chain of the prehelix loop of BMP6—a ligand feature important for type I receptor specificity (Fig. 4, *B* and *C*) (33). Thus, the modeling study is in agreement with previously characterized binding interactions and supports that ERFE may directly occlude the BMP type I receptor site (6).

**Figure 5:**
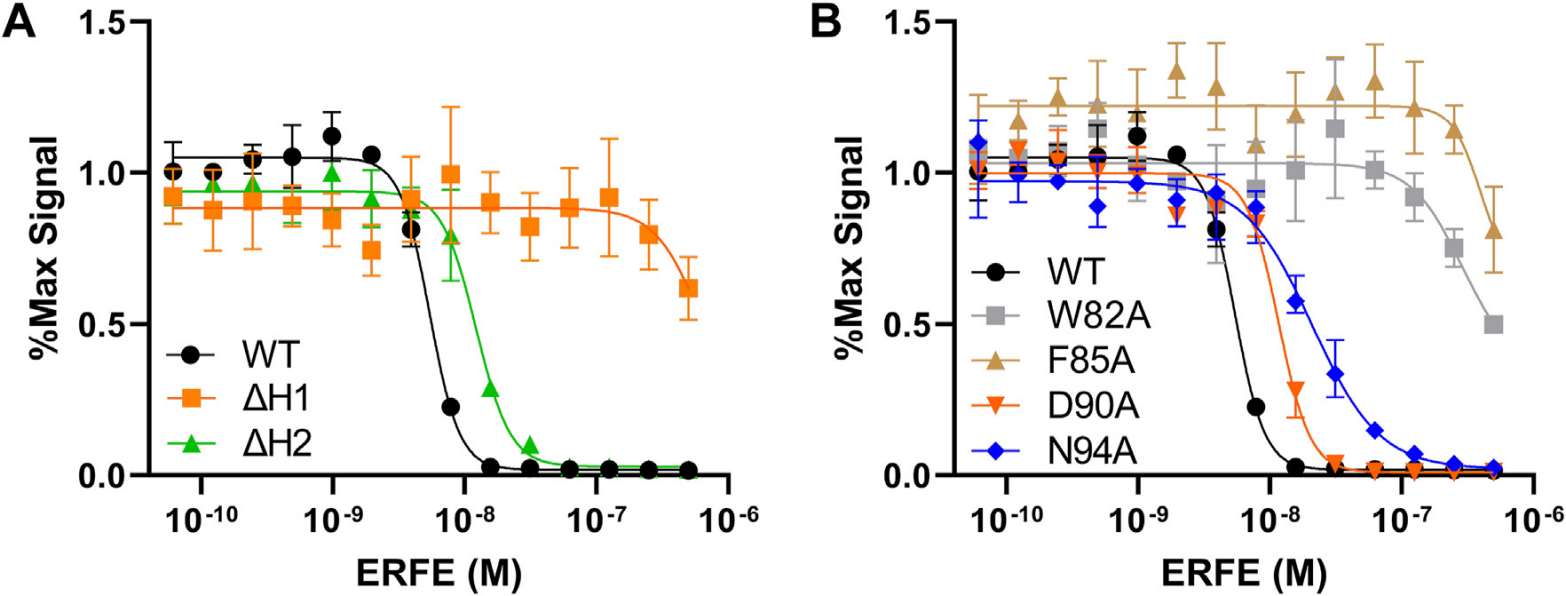
**Mutation of predicted functional ERFE residues in H1 decreased inhibition of BMP6.***A*, removal of predicted interacting helix H1 dramatically reduced BMP inhibition in a luciferase assay, but removal of H2 did not. *B*, mutation of predicted interacting bulky hydrophobic residues in H1 greatly decreased ERFE inhibitory activity, while mutation of predicted interacting charged residues did not. Statistical analysis is detailed in Experimental procedures and results are in Table 3. SD of three points within a single technical replicate shown with error bars. (n = 3 biological replicates, representative curve shown). BMP, bone morphogenetic protein; ERFE, erythroferrone.

**Supporting Figure S4:**
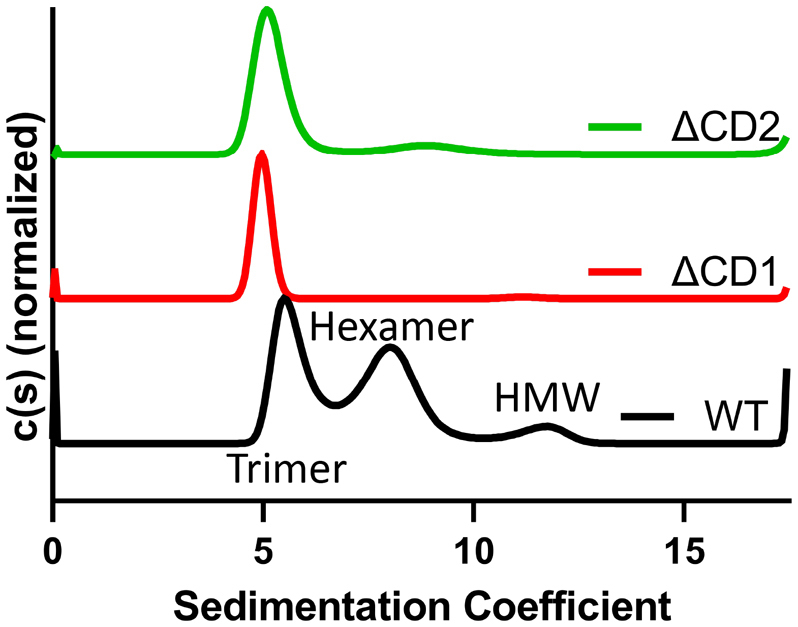
**Oligomeric states of ΔH1, ΔH2 ERFE.** Sedimentation velocity experiments revealed that both ΔH1 and ΔH2 ERFE were deficient in the formation of higher-order ERFE oligomers, but these mutations did not disrupt trimerization. ERFE, erythroferrone.

Based on these results, we designed mutants to test these predicted interactions. We first removed both the H1 and H2 helices. Removal of H1, the helix predicted to interact with BMPs, ablated ERFE inhibition of BMP signaling, while removal of H2 did not impact ERFE potency (Fig. 5*A* and Table 3). Importantly, this was not due to ablation of ERFE trimerization (Fig. S4) Next, we wanted to determine if single point mutations in H1 could impact BMP inhibition. We selected four of the residues predicted to have significant contact with BMP6 in our model and mutated them to alanine—W82A, F85A, D90A, and N94A. Again, recombinant proteins were expressed in Expi293 cells and purified to homogeneity (Fig. S2, *E*–*G*). Strikingly, almost all BMP inhibitory activity was removed by mutations W82A and F85A (Fig. 5*B* and Table 3). D90A and N94A also decreased BMP inhibition, but to a much lesser degree: roughly a 2.5- and 4-fold loss of activity, respectively. Taken together, our data support the BMP6:ERFE-binding interaction predicted by AlphaFold. As H1, the predicted binding component of ERFE’s USD, was critical for activity, we termed it the LBD.

### ΔLBD and monomeric ERFE are less potent inhibitors of ALK3:BMP6 binding

**Figure 6:**
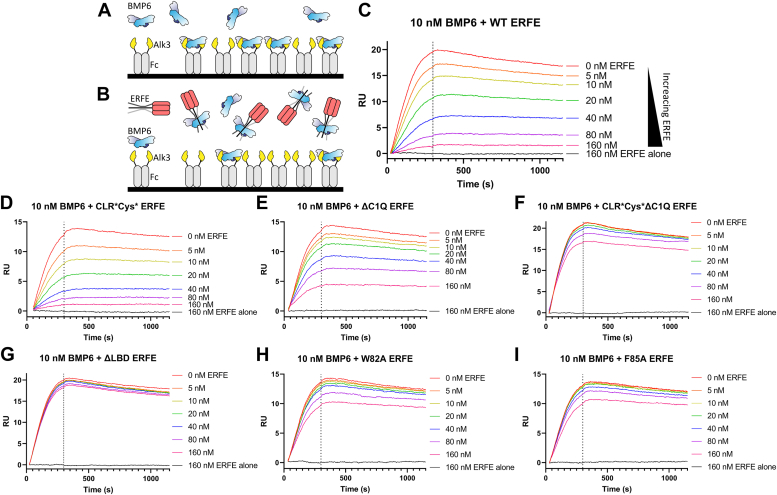
**ΔLBD and monomeric ERFE are less potent inhibitors of ALK3:BMP6 binding.***A*, to analyze ERFE inhibition of BMP:receptor binding, an Alk3-Fc fusion protein was coupled to a SPR protein a chip and 10 nM BMP6 was flowed over. *B*, by flowing over constant BMP6 and increasing the concentration of ERFE, we expected to see less BMP6:Alk3-Fc binding at higher ERFE concentrations. *C*, increasing WT ERFE concentrations decreased BMP6 binding to ALK3. *D*, ablation of ERFE oligomerization above a trimer did not reduce ERFE inhibition of BMP6:Alk3 binding, while (*E* and *F*) ablation of trimerization did. *G*–*I*, ΔLBD ERFE did not prevent BMP6:Alk3 interactions, and this phenotype was partially recapitulated by point mutations of bulky, hydrophobic residues. (n = 2 experimental replicates, representative curve shown). BMP, bone morphogenetic protein; ERFE, erythroferrone; LBD, ligand-binding domain; SPR, surface plasmon resonance.

**Supporting Figure S5:**
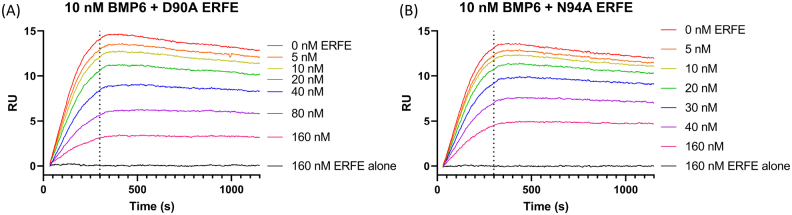
**ERFE D90 and N94 are less important for ERFE inhibition of BMP6:ALK3 binding.***A* and *B*, using the same experimental setup as Figure 6, Alk3-Fc was coupled to a chip and then (*A*) 10 nM BMP6 and D90A ERFE or (*B*) 10 nM BMP6 and N94A ERFE was flowed over and binding was observed. BMP, bone morphogenetic protein; ERFE, erythroferrone.

Finally, we tested the binding model proposed by AlphaFold Multimer to determine if ERFE could disrupt ALK3 binding to BMP6 *via* a competition experiment using SPR (Fig. 6, *A* and *B*). Alk3 was chosen as its affinity for BMP6 is highest among type I receptors, enabling greater sensitivity and lower ligand usage (35). In this experiment, a bivalent Alk3-Fc chimera was captured onto a protein A chip. Coupling density was kept low to avoid nonspecific binding interactions and to reduce the bivalent BMP ligand interacting with multiple Fc molecules. To test if ERFE could block type I receptor interactions, we titrated increasing ERFE with constant BMP6. We predicted ERFE would bind to BMP6 and compete with type I receptor binding, leading to a decrease in response units (Fig. 6, *A* and *B*). BMP6 alone (red) exhibited tight binding to Alk3-Fc, consistent with Isaacs *et. al.*’s reported K_D_ value of 62.46 nM (36) (Fig. 6*C*). ERFE alone (gray) showed no nonspecific binding to Alk3-FC, as expected (6) (Fig. 6*C*). Titrating WT ERFE with constant BMP6 showed a dose-depended decreased response in binding, suggesting disruption of BMP6 binding to Alk3 (Fig. 6*C*). Titration of oligomeric-deficient ERFE mutants revealed decreased inhibition of BMP6:Alk3 interactions mirroring results seen in luciferase assays (Fig. 6, *D*–*F*). Intriguingly, in a similar experiment, ΔLBD ERFE did not have an impact on BMP binding to Alk3, suggesting this mutant is unable to occlude BMP6:Alk3 binding (Fig. 6*G*). ERFE point mutations of bulky hydrophobic residues similarly reduced disruption of BMP6:Alk3 binding, although residual ERFE activity remained (Fig. 6, *H* and *I*). Finally, mutation of charged ERFE LBD residues minimally ablated ERFE activity, confirming our observations in cell-based luciferase reporter assays (Fig. S5, *A* and *B*). These data further support the hypothesis that the ERFE LBD binds to BMP ligands and occludes type I receptor binding.

## Discussion

The role of ERFE in iron regulation is firmly established: both its importance in rapid regeneration of erythrocytes and its contributions to iron overload. Despite this role and its potential other roles in bone growth and metabolic homeostasis (37, 38, 39), its underlying mechanism remained understudied. Prior studies established that ERFE oligomerizes, directly binds to BMP ligands, and suggested it occluded binding to its type 1 receptors. However, neither the impact of oligomerization on activity nor details of this interaction have been examined.

To ascertain the impact of oligomerization on BMP inhibition, we first needed to better characterize WT ERFE and proceeded to define the molecular components responsible for its different states. We found that WT ERFE predominantly forms a trimer and hexamer with a small amount of high-molecular weight species *via* analysis with sedimentation velocity. Mutation of the cysteine residues removes the intermolecular disulfide bonds and ablates both hexamer formation and the high MW species producing a homogenous ERFE trimer. Disruption of the CLR did not alter intermolecular disulfide bond formation but still disrupted formation of hexamer and higher-order oligomers. Thus, ERFE hexamer formation is dependent on both the cysteines and CLR.

Extending these studies to include removal of the C1Q domain helps create a better picture of ERFE oligomerization. Since all our full-length ERFE mutants were trimeric, we predicted the C1Q domain was the primary driver of ERFE trimer formation. However, ΔC1Q was only 67% monomer and the remaining 33% trimerized. The CLR stabilizes ΔC1Q, as mutation disrupted it into monomer and disulfide-linked dimer. Surprisingly, mutation of the cysteines also disrupted residual trimer formation in ΔC1Q, which suggested that interchain disulfide bonds not only play a role in formation of higher-order oligomers but also help stabilize the CLR domain. In fact, the calculated melting temperature of ERFE’s CLR is just 9.1 °C (40). Thus, other interactions may be required to stabilize the expected triple helix of the CLR. By extension, it is possible the lack of hexameric ERFE in full-length Cys∗ is due to the increased instability of the CLR, rather than the lack of intermolecular disulfide bonds. However, additional studies will be required to better characterize mechanisms of higher-order ERFE oligomerization.

Having characterized ERFE mutants that modified oligomeric states, we were better able to understand how oligomerization impacted BMP inhibition. We found that ERFE oligomerization to a hexamer and beyond was dispensable for ERFE inhibition of BMP signaling, while analysis of both WT and mutant ΔC1Q ERFE shows that dimer or trimer formation is indispensable for ERFE activity. Monomeric ERFE (CLR∗Cys∗ΔC1Q) was >35-fold less active than ΔC1Q. The loss in activity is likely due to a loss of avidity. Interestingly, CLR∗ΔC1Q, which does not form a trimer, was only 3-fold less active than ΔC1Q. As CLR∗ΔC1Q still contained disulfide linked dimer, our data suggest that, in the absence of trimer, dimeric ERFE remains active. Consistent with this, Cys∗ΔC1Q, which does not form dimeric species, was just <8% trimer and was 10-fold less active than ΔC1Q. Taken together, these data support that ERFE likely functions as a trimer to inhibit BMP ligands utilizing avidity to maintain a stable inhibitory complex.

**Figure 7:**
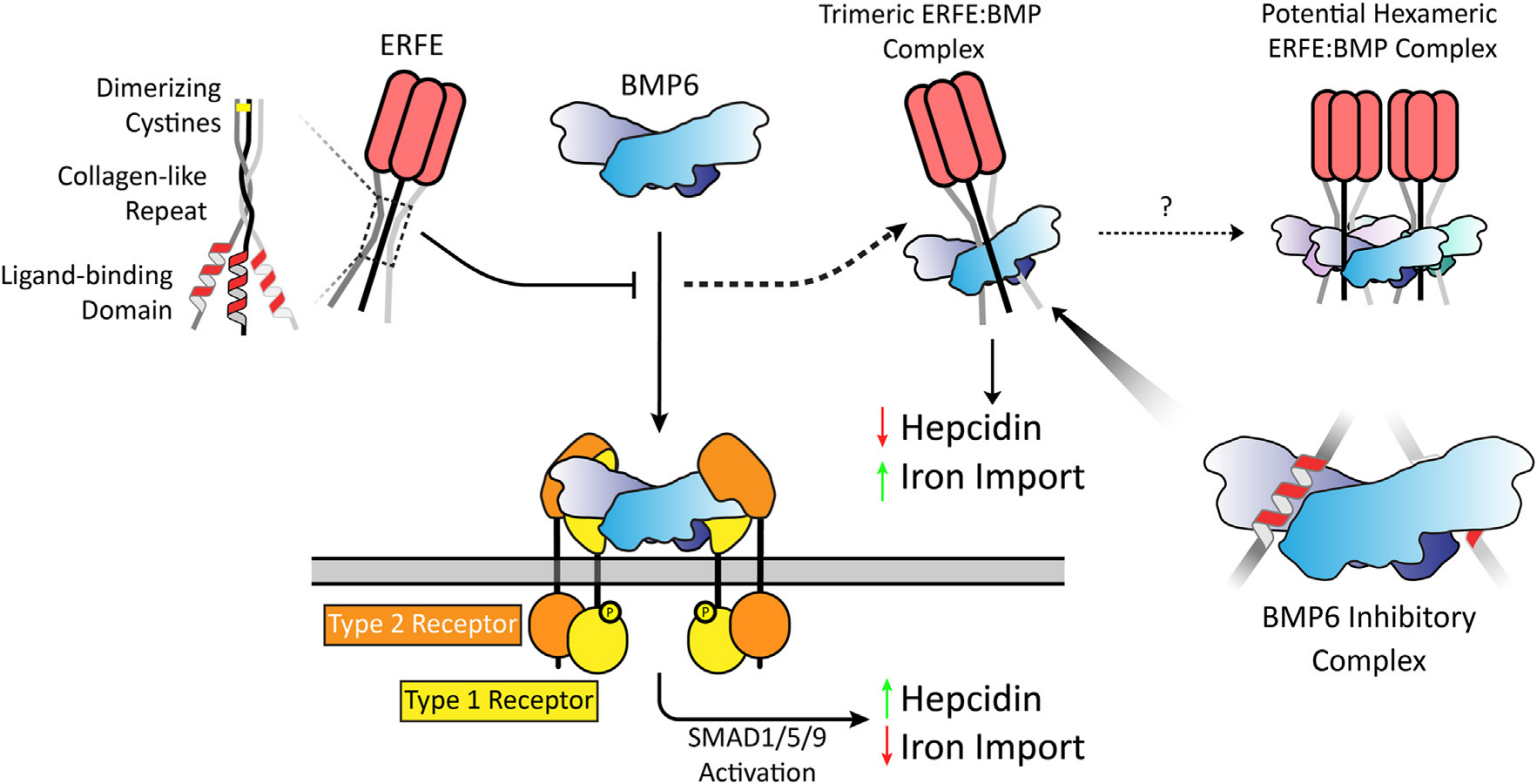
**Trimeric ERFE suppresses hepcidin by binding to BMP6 using its LBD.** The active unit of ERFE is a trimer stabilized by a C1Q domain, interchain disulfide bonds, and CLR. When the body senses iron overload, BMP6 is produced, which induces hepcidin transcription and downregulates iron import. After blood loss, trimeric ERFE negatively regulates this pathway by directly binding and inhibiting BMP6, leading to decreased hepcidin and increased iron import. The BMP6:ERFE interaction is driven by ERFE's conserved BMP-binding helix binding to and occluding the BMP type I receptor site. As ERFE is trimeric and binds to dimeric BMP6, it may either have an unpaired helix or form higher-order bundles using unpaired LBDs. BMP, bone morphogenetic protein; ERFE, erythroferrone; LBD, ligand-binding domain.

Additionally, through a combination of modeling and mutational analysis, we identified a helix in the N terminus of the USD that binds to the type I receptor binding pocket of the ligand. As BMP ligands only exist in the body as disulfide-linked dimers, they possess two type I receptor sites (21, 22). Trimeric ERFE would contain three LBDs, potentially enabling the avidity effects suggested by our ΔC1Q mutant analysis. From these data, we proposed the following model (Fig. 7). Trimeric ERFE, held together by a C1Q domain, an intratrimer disulfide bond, and a collagen-like repeat, binds and inhibits BMP ligands. This downregulates hepcidin induction and upregulates iron import. ERFE does this by occluding BMP ligands’ type I receptor sites through direct binding with its LBDs, which forms a nonsignaling ERFE:BMP complex. Furthermore, this model suggests that trimeric ERFE binds dimeric BMP ligands, potentially leaving an unpaired LBD. Two trimeric ERFE:BMP complexes with unpaired LBDs could potentially pair with a second BMP molecule to form a larger inhibitory complex (Fig. 7). This model would predict that a hexamer of ERFE would be stabilized by binding three BMP ligands. However, support for this model is lacking as attempts to characterize an ERFE:BMP complex are complicated by solubility issues.

**Figure 8:**
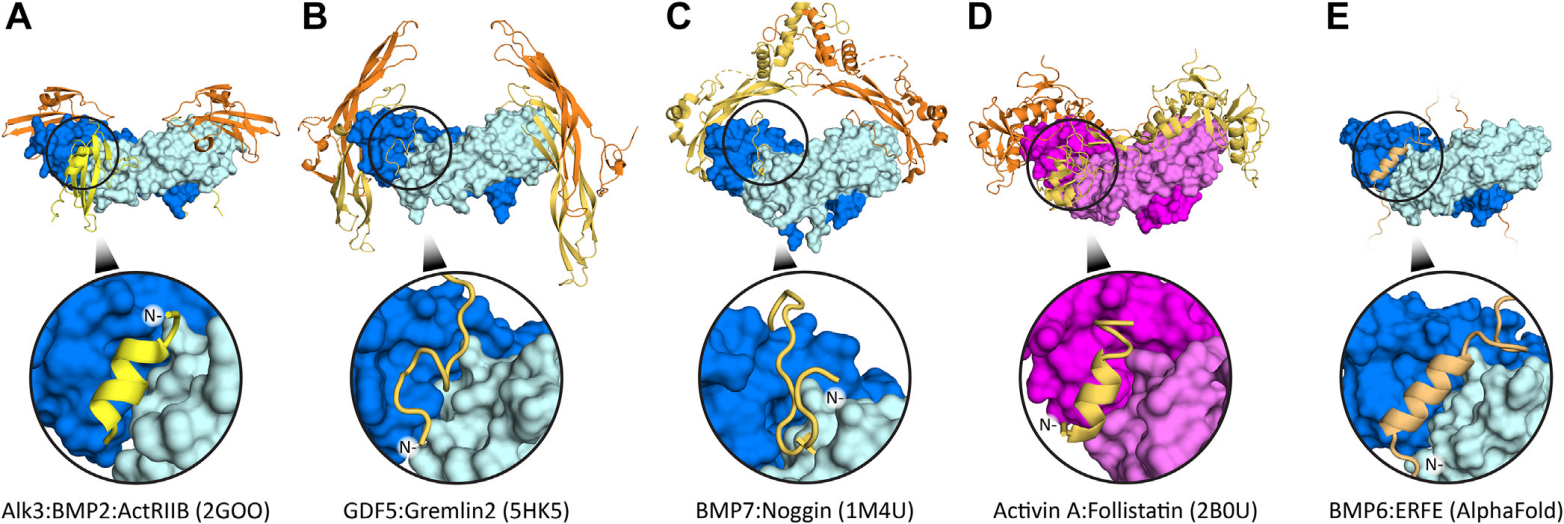
**Structuralcomparisonof TGF-β inhibitors.***A*, type I and type II receptors Alk3 and ActRIIA bind to the concave dimer interface and opposing side of the ligand, respectively. *B* and *C*, Noggin and Gremlin2 both occlude BMP type I receptor binding with n-terminal unstructured loops that form ancillary interactions that complement the large, buried surface area on the opposing side of the ligand. *D*, follistatin interacts with the activin A type I receptor pocket using an n-terminal helix. *E*, while ERFE is currently predicted to interact with the type 1 binding site in a manner similar to other inhibitors, our present model lacks additional interactions with other regions of the ligand. BMP, bone morphogenetic protein; ERFE, erythroferrone.

Comparing ERFE to other extracellular antagonists reveals interesting similarities and differences. BMP ligands bind type I receptors at the concave dimer interface and type II receptors on the convex surface (41). Figure 8 highlights how various inhibitors bind and occlude the type I binding site on the ligand. Noggin and Gremlin form extensive contacts on the knuckle region of the ligand and thread a peptide into the type I site (using a knob-in-hole) (24, 26). Both follistatin and ERFE bind using a more well-ordered helix to bind in the same pocket (42). Other noninhibitory binding partners utilize helical motifs as well; ligand prodomains bind using an α1 helix and the repulsive guidance molecule family, which act as critical BMP coreceptors, interact using a bundle of helices (43, 44). Interestingly, the aforementioned helices and loops in BMP antagonists and binding partners thread through the type I receptor site in the opposite direction as the receptor helices. ERFE may separate itself from other characterized antagonistic interactions which occlude both type I and type II receptor sites, as our current data suggest ERFE only occludes type I receptor binding. Further studies may reveal additional points of contact between ligand and antagonist or show that ERFE’s avidity allows it to circumvent the need for the extensive interactions seen in other inhibitors.

In conclusion, we have demonstrated that the basic functional unit of ERFE is a trimer, as all of its oligomerizing features all stabilize trimer formation. Meanwhile, oligomerization above a trimer is dispensable for inhibition of BMP signaling. Finally, we identified a key conserved segment in ERFE’s USD that is critical for activity and occlusion of the type I receptor site. Whether this is the only molecular feature that interacts with BMP ligands is not known. Future structural studies are required to both validate the modeling efforts and determine the extent of the ERFE:BMP interaction. Furthermore, while our study also highlights the specificity of ERFE for certain BMP ligands, the molecular basis for this specificity remains undetermined.

## Experimental procedures

### Recombinant ligands and detection antibodies

Activin A, anti-Müllerian hormone, BMP2, and GDF5 were all produced as previously described (45, 46, 47). Mature GDF11 was a generous gift from Elevian. BMP6 was a generous gift from OSTEOGROW. ERFE was detected using DYKDDDDK Epitope Tag HRP-conjugated antibody (R&D, Cat. # HAM85291) and FAM132B polyclonal antibody (Invitrogen, Cat. #PA5-67448). Recombinant ALK3-Fc fusion protein was purchased from R&D (Cat. No. 2406-BR-100).

### Protein production and purification

ERFE production was performed using a N-terminally flag-tagged hERFE construct (residues 44–354) in the mammalian expression vector pcDNA3.1 generously given by Tomas Ganz (UCLA) (48). Purified plasmid was transfected into Expi293 cells at density 2.0 × 10^6^ using polyethylenimine. Media were collected at day 3 applied directly to a HiPrep Heparin FF affinity column. Protein was eluted with a gradient from 500 to 650 mM NaCl at pH7.5 with 20 mM Hepes and 5 mM CaCl2. A single peak containing ERFE was pooled and dialyzed into 20 mM Hepes pH 7.5, 150 mM NaCl, and 5 mM CaCl_2_, concentrated to 1 mg/ml and either used immediately or flash frozen. WT yields ranged between 1 and 2 mg/L and varied mutant to mutant.

For ERFE mutants that were unable to be purified solely *via* heparin affinity, conditioned media were first applied to αFlag resin and eluted with 100 mM glycine pH 2.5. After neutralization with 500 mM Hepes pH 7.5, it was applied to a heparin column and purified in the same manner described above. Yields for this preparatory method were slightly lower than exclusively using heparin affinity chromatography. No change in activity was seen when WT ERFE was purified using this method (Fig. S1*C*).

BMP7 was produced using a modified protocol from Cate *et al.* (49) In summary, BMP7 cDNA in a pRK5 mammalian expression vector was transfected into Expi293 cells with polyethylenimine. Conditioned media were harvested 3 days posttransfection and applied to a HiTrap SP Sepharose cation exchange column at pH7.5 and eluted using a salt gradient (Hepes pH 7.5, 0–1 M NaCl, 1 mM EDTA). Fractions were pooled and dialyzed into 4 mM HCl overnight before being loaded onto a reversed phase C18 column, eluted with an acetonitrile gradient, and flash frozen after dialysis into 4 mM HCl. Average yields were approximately 0.35 mg mature ligand per liter conditioned media. Activity was confirmed *via* luciferase reporter assay in BRITER cells (Fig. S1*D*) (50).

### Luciferase reporter assay

A BMP-responsive luciferase reporter osteoblast cell line (BRITER, RRID:CVCL_0P40), provided by Amitabha Bandyopadhyay (Indian Institute of Technology), was used to measure BMP activity as previously reported (50, 51, 52). Briefly, cells were grown overnight in α-minimal essential medium with 10% (v/v) FBS and 100 μg/ml hygromycin B in a 96-well plate at 37 °C in 5% CO2. The medium was replaced with Dulbecco's modified Eagle's medium, and cells were starved for 5 h. Activity assays were performed by titrating ligands in Dulbecco's modified Eagle's medium, while inhibition assays were performed by titrating inhibitor and adding constant ligand. The media were replaced with ligand or ligand+inhibitor and incubated at the same conditions for 3 h before luminescence was read using a BioTek Synergy H1 plate reader. Inhibitory curves were normalized using ligand alone as 100% signal. Curve fits and both EC_50_ and IC_50_ values were generated using GraphPad Prism. All IC_50_ values were calculated using a variable hill slope. Each experiment consisted of three technical replicates, and either two or three biological replicates were performed, as indicated. Error bars on graphs represent the SD of the three technical replicates from the representative sample. Reported IC_50_ are the average of three biological replicates ± SEM.

### Analytical ultracentrifugation

Protein samples were used postdialysis and were never frozen. Sedimentation velocity experiments were run using Beckman Optima XL-I analytical ultracentrifuge (Beckman Coulter), An60-Ti rotor, and absorbance optics. Samples at 0.5 mg/ml were loaded into Beckman AUC sample cells with 12-mm optical path two-channel centerpieces, with matched buffer in the reference sector. Full-length ERFE constructs were centrifugated at 20,000 rpm, while ΔC1Q mutants were centrifugated at 40,000 RPM. Absorbance was measured at 230 nm to maximize signal, and the experiments were performed at 20 °C. SEDFIT was used to determine a preliminary frictional ratio using continuous c(s,ff0) distribution. The most commonly occurring frictional ratio was used as a starting value to solve for a precise frictional ratio using c(s) distributions, which yielded predicted molecular weights. In rare cases where solved c(s) and c(s,ff0) frictional ratios diverged significantly, the three most frequent c(s,ff0) frictional ratio were averaged, which ameliorated the issue. All datasets were normalized on a scale from 0 to 1, using the highest and lowest value.

### AlphaFold and Consurf

AlphaFold 2.3.0 was used to create models of the ERFE:BMP6 complex. Five AMBER relaxed models were generated. Analysis was focused on the top ranked model. Potential interactions were analyzed using PDBePISA (53) and PyMOL (54). For analysis of evolutionary conservation, ERFE (AA 29–354) was analyzed using the ConSurf webserver with default settings (29, 30). Results were used to examine conservation of predicted interacting residues before mutagenesis.

### Surface plasmon resonance

Binding kinetics of BMP6 to Alk3 was determined by SPR using a BIAcore T-200 optical sensor system. 0.45 μg/ml Alk3-Fc was first captured on a protein a chip (Cat. # 29127555), establishing a baseline of 85 response units. 10 nM constant BMP6 with ERFE concentrations serially diluted in SPR running buffer (20 mM Hepes pH 7.4, 350 mM NaCl, 0.005% Tween-20, 0.5 mg/ml BSA, 5 mM EDTA) to 160 to 5 nM, were flowed over the chip at 50 μl/min for 300 s to observe association, then washed off for 900 s to observe dissociation. Additionally, ERFE alone at 160 nM was flowed over the chip as a control. Regeneration was performed using 10 mM glycine pH 1.8.

## Data availability

All data are available upon request.

## Supporting information

This article contains supporting information.

## Conflict of interest

T. B. T. is a consultant/advisor for Keros Therapeutics and Oviva Therapeutics. All other authors declare they have no conflicts of interest with the contents of this article.
